# 5-Methyl­spiro­[indoline-3,7′-[6*H*,7*H*,8*H*]pyrano[3,2-*c*:5,6-*c*′]di[1]benzopyran]-2,6′,8′-trione chloroform hemisolvate

**DOI:** 10.1107/S1600536812012020

**Published:** 2012-03-28

**Authors:** Abdulrahman I. Almansour, Raju Suresh Kumar, Natarajan Arumugam, R. Vishnupriya, J. Suresh

**Affiliations:** a Department of Chemistry, College of Sciences, King Saud University, PO Box 2455, Riyadh 11451, Saudi Arabia; b Department of Physics, The Madura College, Madurai 625 011, India

## Abstract

In the title compound, C_27_H_15_NO_6_·0.5CHCl_3_, the central pyran ring and both the benzopyran systems are planar, with the dihedral angle between the outer rings being 3.24 (6)°. The indolin-2-one system is in a perpendicular configuration with respect to the pyran ring [dihedral angle = 87.58 (2)°]. Supra­molecular layers in the *ac* plane are formed in the crystal structure whereby inversion-related mol­ecules are connected by N—H⋯O hydrogen bonds. These are further linked by C—H⋯O inter­actions, forming a supra­molecular layer in the *ac* plane. Disordered CHCl_3_ solvent in the structure was modelled with the SQUEEZE routine in *PLATON* [Spek (2009 ▶). *Acta Cryst.* D**65**, 148–155].

## Related literature

For hydrogen-bonding motifs, see: Bernstein *et al.* (1995 ▶). For the biological relevance of benzopyrans, see: Martin & Critchlow (1999 ▶); Teague & Davis (1999 ▶). For the importance of spiro­[indole-pyran] systems, see: Ninamiya (1980 ▶); Kobayashi & Matsuda (1970 ▶).
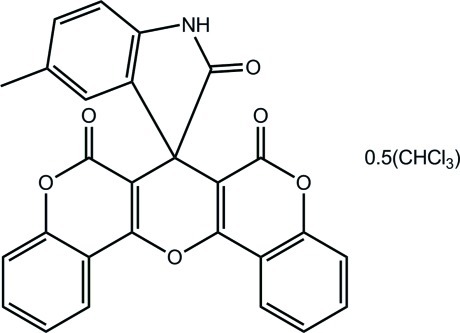


## Experimental

### 

#### Crystal data

C_27_H_15_NO_6_·0.5CHCl_3_
*M* *_r_* = 509.08
Monoclinic, 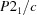

*a* = 9.9341 (2) Å
*b* = 19.1498 (4) Å
*c* = 12.8279 (2) Å
β = 95.078 (1)°
*V* = 2430.75 (8) Å^3^
*Z* = 4
Mo *K*α radiation
μ = 0.26 mm^−1^
*T* = 293 K
0.21 × 0.17 × 0.12 mm

#### Data collection

Bruker Kappa APEXII diffractometer
Absorption correction: multi-scan (*SADABS*; Sheldrick, 1996 ▶) *T*_min_ = 0.973, *T*_max_ = 0.978
30555 measured reflections
7646 independent reflections
5443 reflections with *I* > 2σ(*I*)
*R*_int_ = 0.038

#### Refinement

*R*[*F* ^2^ > 2σ(*F* ^2^)] = 0.067
*wR*(*F* ^2^) = 0.174
*S* = 1.05
7646 reflections
312 parameters
H atoms treated by a mixture of independent and constrained refinement
Δρ_max_ = 0.56 e Å^−3^
Δρ_min_ = −0.26 e Å^−3^

### 

Data collection: *APEX2* (Bruker, 2004 ▶); cell refinement: *SAINT* (Bruker, 2004 ▶); data reduction: *SAINT*; program(s) used to solve structure: *SHELXS97* (Sheldrick, 2008 ▶); program(s) used to refine structure: *SHELXL97* (Sheldrick, 2008 ▶); molecular graphics: *PLATON* (Spek, 2009 ▶); software used to prepare material for publication: *SHELXL97*.

## Supplementary Material

Crystal structure: contains datablock(s) global, I. DOI: 10.1107/S1600536812012020/tk5070sup1.cif

Structure factors: contains datablock(s) I. DOI: 10.1107/S1600536812012020/tk5070Isup2.hkl

Additional supplementary materials:  crystallographic information; 3D view; checkCIF report

## Figures and Tables

**Table 1:** Hydrogen-bond geometry (Å, °)

*D*—H⋯*A*	*D*—H	H⋯*A*	*D*⋯*A*	*D*—H⋯*A*
N1—H1⋯O5^i^	0.89 (3)	2.25 (3)	2.965 (2)	138 (2)
C45—H45⋯O5^i^	0.93	2.51	3.212 (2)	133
C63—H63⋯O3^ii^	0.93	2.51	3.347 (3)	150
C65—H65⋯O4^iii^	0.93	2.59	3.363 (2)	141

Symmetry codes: (i) 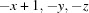; (ii) 
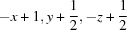
; (iii) 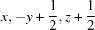.

**Fig. 1.**
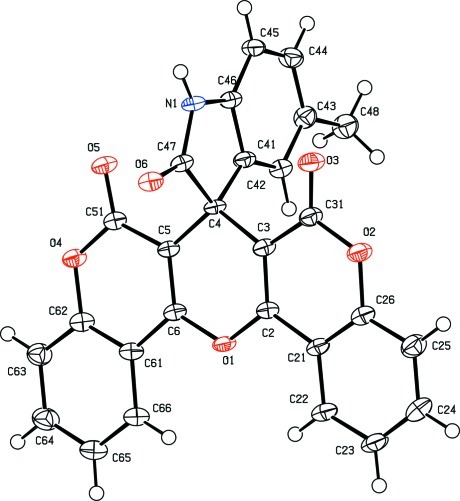
The molecular structure of (I), showing 50% probability displacement ellipsoids and the atom-numbering scheme.

**Fig. 2.**
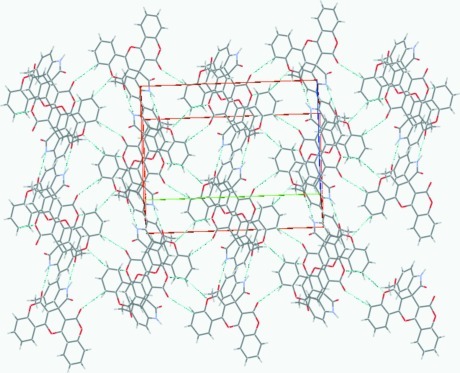
A packing diagram for (I).

## supplementary crystallographic information

### Comment

Benzopyran is a structural motif observed in many biologically active natural products and it plays an important role in binding with various biopolymers (Martin *et al.*, 1999; Teague *et al.*, 1999). Of the various spiro indoles, the spiro[indole-pyran] system has attracted attention due to its interesting pharmacological properties (Ninamiya *et al.*, 1980; Kobayashi *et al.*, 1970). The biological importance of these heterocycles in conjunction with our research interests prompted us to synthesize and report the X-ray structure of the title compound.

In the title compound, Fig 1, the central pyrano ring and both the benzopyran rings are planar. In the indolin-2-one system, the benzene and pyrrole rings are individually planar and make a dihedral angle of 1.42 (1)°. The indoline-2-one system is in a perpendicular configuration with respect to the pyrano ring, as can be seen from the dihedral angle of 87.58 (2)°. The sum of the angles at atom N1 of the indolin-2-one moiety is in accordance with *sp*^2^-hybridization [359.85 (2)°].

The N1—H1···O5 hydrogen bond connects two centrosymmetrically related molecules and generate the graph set motif *R*_2_^2^(14) (Bernstein *et al.*, 1995). These centrosymmetric dimers are interrelated by zigzag linear chains of C—H···O hydrogen bonds to form a layered structure (Fig. 2).

### Experimental

A mixture of 5-methylindoline-2,3-dione (0.100 g, 0.62 mmol), 4-hydroxy-2*H*-chromen-2-one (0.201 g, 1.24 mmol) and paratoluene sulfonic acid (0.118 g, 0.62 mmol) were dissolved in 5 ml of ethanol:water (1:1 *v*/*v*) and refluxed for 2 h. After completion of the reaction, as evidenced from TLC, the precipitated solid was filtered and washed with water to afford the product which was recrystallized from CHCl_3_ to produce the title compound as colourless crystals. Yield 76%. *M*. pt: 540–541 K.

### Refinement

Initial structural solution showed a disordered co-crystallized solvent chloroform molecule for which a suitable model could not be found. Therefore, the data set was treated with SQUEEZE routine of *PLATON* (Spek, 2009) to model the electron density in the void regions. There are two cavities of 277 Å^3^ per unit cell. Each cavity contains approximately 125 electrons which were assigned to two solvent chloroform molecules. The N-bound H atom was located in a difference map and refined freely. The C-bound H atoms were placed at calculated positions and allowed to ride on their carrier atoms with C—H = 0.93–0.96 Å, and with *U*_iso_ = 1.2 to 1.5*U*_eq_(C).

### Crystal data

**Table:**

C_27_H_15_NO_6_·0.5CHCl_3_	*F*(000) = 1044
*M**_r_* = 509.08	*D*_x_ = 1.391 Mg m^−^^3^
Monoclinic, *P*2_1_/*c*	Mo *K*α radiation, λ = 0.71073 Å
Hall symbol: -P 2ybc	Cell parameters from 2000 reflections
*a* = 9.9341 (2) Å	θ = 2–31°
*b* = 19.1498 (4) Å	µ = 0.26 mm^−^^1^
*c* = 12.8279 (2) Å	*T* = 293 K
β = 95.078 (1)°	Block, colourless
*V* = 2430.75 (8) Å^3^	0.21 × 0.17 × 0.12 mm
*Z* = 4	

### Data collection

**Table:**

Bruker Kappa APEXII diffractometer	7646 independent reflections
Radiation source: fine-focus sealed tube	5443 reflections with *I* > 2σ(*I*)
Graphite monochromator	*R*_int_ = 0.038
Detector resolution: 0 pixels mm^-1^	θ_max_ = 31.0°, θ_min_ = 1.9°
ω and φ scans	*h* = −14→13
Absorption correction: multi-scan (*SADABS*; Sheldrick, 1996)	*k* = −25→27
*T*_min_ = 0.973, *T*_max_ = 0.978	*l* = −18→18
30555 measured reflections	

### Refinement

**Table:**

Refinement on *F*^2^	Primary atom site location: structure-invariant direct methods
Least-squares matrix: full	Secondary atom site location: difference Fourier map
*R*[*F*^2^ > 2σ(*F*^2^)] = 0.067	Hydrogen site location: inferred from neighbouring sites
*wR*(*F*^2^) = 0.174	H atoms treated by a mixture of independent and constrained refinement
*S* = 1.05	*w* = 1/[σ^2^(*F*_o_^2^) + (0.0634*P*)^2^ + 2.2541*P*] where *P* = (*F*_o_^2^ + 2*F*_c_^2^)/3
7646 reflections	(Δ/σ)_max_ < 0.001
312 parameters	Δρ_max_ = 0.56 e Å^−^^3^
0 restraints	Δρ_min_ = −0.26 e Å^−^^3^

### Special details

**Table:**

Geometry. All e.s.d.'s (except the e.s.d. in the dihedral angle between two l.s. planes) are estimated using the full covariance matrix. The cell e.s.d.'s are taken into account individually in the estimation of e.s.d.'s in distances, angles and torsion angles; correlations between e.s.d.'s in cell parameters are only used when they are defined by crystal symmetry. An approximate (isotropic) treatment of cell e.s.d.'s is used for estimating e.s.d.'s involving l.s. planes.
Refinement. Refinement of *F*^2^ against ALL reflections. The weighted *R*-factor *wR* and goodness of fit *S* are based on *F*^2^, conventional *R*-factors *R* are based on *F*, with *F* set to zero for negative *F*^2^. The threshold expression of *F*^2^ > σ(*F*^2^) is used only for calculating *R*-factors(gt) *etc*. and is not relevant to the choice of reflections for refinement. *R*-factors based on *F*^2^ are statistically about twice as large as those based on *F*, and *R*- factors based on ALL data will be even larger.

### Fractional atomic coordinates and isotropic or equivalent isotropic displacement parameters (Å^2^)

**Table:**

	*x*	*y*	*z*	*U*_iso_*/*U*_eq_	
H1	0.457 (3)	−0.0749 (15)	0.036 (2)	0.039 (7)*	
O1	0.36729 (14)	0.05045 (7)	0.45866 (9)	0.0208 (3)	
O4	0.61175 (14)	0.16085 (7)	0.27455 (10)	0.0218 (3)	
O2	0.13685 (15)	−0.11955 (8)	0.35920 (10)	0.0260 (3)	
O5	0.55602 (15)	0.09408 (7)	0.13736 (10)	0.0237 (3)	
C5	0.45433 (18)	0.06556 (9)	0.29103 (13)	0.0173 (3)	
C4	0.37191 (18)	0.00753 (9)	0.23811 (12)	0.0169 (3)	
O3	0.21718 (16)	−0.11890 (8)	0.20426 (11)	0.0274 (3)	
C61	0.5243 (2)	0.14056 (10)	0.44128 (13)	0.0206 (3)	
O6	0.55858 (14)	−0.07739 (7)	0.22758 (10)	0.0227 (3)	
N1	0.42069 (17)	−0.04625 (8)	0.08013 (11)	0.0194 (3)	
C51	0.54180 (19)	0.10595 (9)	0.22880 (13)	0.0191 (3)	
C62	0.6029 (2)	0.17960 (10)	0.37751 (14)	0.0224 (4)	
C21	0.20946 (19)	−0.03669 (10)	0.49302 (13)	0.0201 (3)	
C26	0.1330 (2)	−0.09402 (10)	0.45856 (14)	0.0232 (4)	
C6	0.44858 (19)	0.08330 (9)	0.39331 (13)	0.0187 (3)	
C66	0.5210 (2)	0.16042 (10)	0.54660 (14)	0.0242 (4)	
H66	0.4702	0.1348	0.5907	0.029*	
C31	0.2182 (2)	−0.09080 (10)	0.28832 (14)	0.0223 (4)	
C2	0.29255 (19)	−0.00495 (9)	0.41954 (13)	0.0189 (3)	
C3	0.29520 (19)	−0.02882 (10)	0.31997 (13)	0.0201 (3)	
C22	0.2006 (2)	−0.01261 (11)	0.59664 (14)	0.0255 (4)	
H22	0.2501	0.0261	0.6213	0.031*	
C47	0.46395 (19)	−0.04504 (9)	0.18295 (13)	0.0194 (3)	
C41	0.27664 (19)	0.03172 (10)	0.14649 (13)	0.0199 (3)	
C23	0.1178 (2)	−0.04734 (12)	0.66075 (15)	0.0297 (4)	
H23	0.1116	−0.0318	0.7289	0.036*	
C65	0.5931 (2)	0.21793 (11)	0.58413 (16)	0.0285 (4)	
H65	0.5922	0.2307	0.6540	0.034*	
C42	0.17032 (19)	0.07831 (10)	0.14353 (14)	0.0214 (4)	
H42	0.1506	0.1013	0.2042	0.026*	
C63	0.6734 (2)	0.23846 (10)	0.41314 (16)	0.0262 (4)	
H63	0.7229	0.2647	0.3689	0.031*	
C44	0.1267 (2)	0.05638 (11)	−0.04015 (15)	0.0260 (4)	
H44	0.0752	0.0646	−0.1031	0.031*	
C25	0.0495 (2)	−0.12924 (12)	0.52263 (16)	0.0298 (4)	
H25	−0.0008	−0.1677	0.4979	0.036*	
C45	0.2353 (2)	0.00990 (10)	−0.03905 (14)	0.0230 (4)	
H45	0.2569	−0.0123	−0.0999	0.028*	
C46	0.30960 (19)	−0.00194 (10)	0.05576 (13)	0.0196 (3)	
C43	0.0926 (2)	0.09085 (10)	0.04953 (15)	0.0244 (4)	
C24	0.0432 (2)	−0.10568 (13)	0.62359 (17)	0.0332 (5)	
H24	−0.0114	−0.1288	0.6677	0.040*	
C48	−0.0246 (2)	0.14117 (12)	0.04545 (17)	0.0301 (4)	
H48A	−0.0772	0.1368	−0.0207	0.045*	
H48B	0.0090	0.1880	0.0535	0.045*	
H48C	−0.0802	0.1307	0.1010	0.045*	
C64	0.6678 (2)	0.25713 (11)	0.51757 (17)	0.0306 (4)	
H64	0.7145	0.2964	0.5435	0.037*	

### Atomic displacement parameters (Å^2^)

**Table:**

	*U*^11^	*U*^22^	*U*^33^	*U*^12^	*U*^13^	*U*^23^
O1	0.0288 (7)	0.0230 (6)	0.0110 (5)	−0.0020 (5)	0.0047 (5)	−0.0020 (5)
O4	0.0298 (7)	0.0208 (6)	0.0154 (6)	−0.0013 (5)	0.0046 (5)	0.0001 (5)
O2	0.0354 (8)	0.0263 (7)	0.0173 (6)	−0.0067 (6)	0.0078 (5)	−0.0023 (5)
O5	0.0338 (8)	0.0235 (7)	0.0147 (6)	−0.0001 (6)	0.0070 (5)	−0.0009 (5)
C5	0.0231 (8)	0.0167 (7)	0.0124 (7)	0.0018 (6)	0.0026 (6)	0.0006 (6)
C4	0.0244 (8)	0.0167 (7)	0.0098 (6)	0.0006 (6)	0.0039 (6)	−0.0007 (5)
O3	0.0395 (8)	0.0252 (7)	0.0183 (6)	−0.0056 (6)	0.0074 (6)	−0.0041 (5)
C61	0.0277 (9)	0.0198 (8)	0.0140 (7)	0.0028 (7)	0.0011 (7)	−0.0004 (6)
O6	0.0288 (7)	0.0223 (6)	0.0172 (6)	0.0041 (5)	0.0034 (5)	0.0003 (5)
N1	0.0274 (8)	0.0203 (7)	0.0110 (6)	0.0028 (6)	0.0046 (5)	−0.0018 (5)
C51	0.0243 (9)	0.0181 (8)	0.0152 (7)	0.0025 (7)	0.0031 (6)	0.0004 (6)
C62	0.0289 (10)	0.0215 (8)	0.0170 (8)	0.0018 (7)	0.0028 (7)	−0.0007 (6)
C21	0.0245 (9)	0.0233 (8)	0.0132 (7)	0.0034 (7)	0.0048 (6)	0.0015 (6)
C26	0.0273 (9)	0.0262 (9)	0.0168 (8)	0.0021 (7)	0.0057 (7)	0.0006 (7)
C6	0.0247 (9)	0.0186 (8)	0.0129 (7)	0.0022 (7)	0.0031 (6)	0.0009 (6)
C66	0.0315 (10)	0.0254 (9)	0.0158 (8)	0.0011 (8)	0.0019 (7)	−0.0023 (7)
C31	0.0278 (9)	0.0231 (9)	0.0165 (8)	−0.0016 (7)	0.0052 (7)	0.0001 (6)
C2	0.0242 (9)	0.0195 (8)	0.0132 (7)	0.0013 (7)	0.0023 (6)	0.0001 (6)
C3	0.0257 (9)	0.0205 (8)	0.0145 (7)	0.0001 (7)	0.0049 (6)	−0.0006 (6)
C22	0.0318 (10)	0.0304 (10)	0.0148 (8)	0.0033 (8)	0.0058 (7)	−0.0001 (7)
C47	0.0263 (9)	0.0185 (8)	0.0143 (7)	0.0005 (7)	0.0059 (6)	−0.0009 (6)
C41	0.0257 (9)	0.0222 (8)	0.0123 (7)	−0.0013 (7)	0.0038 (6)	−0.0002 (6)
C23	0.0351 (11)	0.0391 (11)	0.0162 (8)	0.0029 (9)	0.0091 (7)	−0.0001 (8)
C65	0.0365 (11)	0.0289 (10)	0.0198 (8)	0.0008 (9)	0.0015 (8)	−0.0059 (7)
C42	0.0255 (9)	0.0227 (8)	0.0165 (8)	0.0011 (7)	0.0046 (7)	−0.0007 (6)
C63	0.0314 (10)	0.0232 (9)	0.0240 (9)	−0.0021 (8)	0.0021 (8)	−0.0008 (7)
C44	0.0295 (10)	0.0300 (10)	0.0181 (8)	−0.0010 (8)	−0.0003 (7)	0.0008 (7)
C25	0.0347 (11)	0.0304 (10)	0.0254 (9)	−0.0035 (9)	0.0091 (8)	0.0007 (8)
C45	0.0277 (9)	0.0275 (9)	0.0139 (7)	−0.0019 (8)	0.0024 (7)	−0.0023 (6)
C46	0.0253 (9)	0.0199 (8)	0.0141 (7)	−0.0004 (7)	0.0044 (6)	−0.0016 (6)
C43	0.0249 (9)	0.0254 (9)	0.0230 (9)	0.0009 (7)	0.0039 (7)	0.0020 (7)
C24	0.0362 (12)	0.0407 (12)	0.0248 (10)	−0.0017 (10)	0.0138 (8)	0.0036 (9)
C48	0.0289 (10)	0.0342 (11)	0.0272 (10)	0.0065 (9)	0.0031 (8)	0.0020 (8)
C64	0.0372 (12)	0.0273 (10)	0.0271 (10)	−0.0036 (9)	0.0013 (9)	−0.0068 (8)

### Geometric parameters (Å, º)

**Table:**

O1—C2	1.365 (2)	C31—C3	1.451 (3)
O1—C6	1.368 (2)	C2—C3	1.359 (2)
O4—C51	1.364 (2)	C22—C23	1.384 (3)
O4—C62	1.379 (2)	C22—H22	0.9300
O2—C26	1.369 (2)	C41—C42	1.381 (3)
O2—C31	1.383 (2)	C41—C46	1.395 (2)
O5—C51	1.215 (2)	C23—C24	1.401 (3)
C5—C6	1.361 (2)	C23—H23	0.9300
C5—C51	1.454 (2)	C65—C64	1.398 (3)
C5—C4	1.506 (2)	C65—H65	0.9300
C4—C41	1.515 (2)	C42—C43	1.394 (3)
C4—C3	1.520 (2)	C42—H42	0.9300
C4—C47	1.570 (2)	C63—C64	1.392 (3)
O3—C31	1.204 (2)	C63—H63	0.9300
C61—C62	1.396 (3)	C44—C43	1.394 (3)
C61—C66	1.407 (2)	C44—C45	1.398 (3)
C61—C6	1.437 (3)	C44—H44	0.9300
O6—C47	1.225 (2)	C25—C24	1.378 (3)
N1—C47	1.351 (2)	C25—H25	0.9300
N1—C46	1.405 (2)	C45—C46	1.384 (3)
N1—H1	0.88 (3)	C45—H45	0.9300
C62—C63	1.384 (3)	C43—C48	1.509 (3)
C21—C26	1.385 (3)	C24—H24	0.9300
C21—C22	1.417 (2)	C48—H48A	0.9600
C21—C2	1.441 (2)	C48—H48B	0.9600
C26—C25	1.393 (3)	C48—H48C	0.9600
C66—C65	1.378 (3)	C64—H64	0.9300
C66—H66	0.9300		
			
C2—O1—C6	117.62 (13)	C21—C22—H22	120.3
C51—O4—C62	122.64 (15)	O6—C47—N1	127.77 (17)
C26—O2—C31	122.42 (16)	O6—C47—C4	124.55 (15)
C6—C5—C51	118.32 (16)	N1—C47—C4	107.67 (15)
C6—C5—C4	123.45 (16)	C42—C41—C46	120.75 (17)
C51—C5—C4	118.21 (14)	C42—C41—C4	130.01 (16)
C5—C4—C41	113.67 (14)	C46—C41—C4	109.24 (16)
C5—C4—C3	108.20 (13)	C22—C23—C24	120.37 (18)
C41—C4—C3	111.05 (15)	C22—C23—H23	119.8
C5—C4—C47	111.18 (15)	C24—C23—H23	119.8
C41—C4—C47	101.25 (13)	C66—C65—C64	120.24 (18)
C3—C4—C47	111.43 (14)	C66—C65—H65	119.9
C62—C61—C66	118.68 (17)	C64—C65—H65	119.9
C62—C61—C6	117.14 (16)	C41—C42—C43	119.94 (17)
C66—C61—C6	124.15 (17)	C41—C42—H42	120.0
C47—N1—C46	112.50 (15)	C43—C42—H42	120.0
C47—N1—H1	121.3 (18)	C62—C63—C64	117.69 (19)
C46—N1—H1	126.0 (18)	C62—C63—H63	121.2
O5—C51—O4	117.46 (16)	C64—C63—H63	121.2
O5—C51—C5	123.83 (17)	C43—C44—C45	122.47 (18)
O4—C51—C5	118.71 (15)	C43—C44—H44	118.8
O4—C62—C63	117.07 (17)	C45—C44—H44	118.8
O4—C62—C61	120.54 (17)	C24—C25—C26	118.4 (2)
C63—C62—C61	122.39 (17)	C24—C25—H25	120.8
C26—C21—C22	118.67 (17)	C26—C25—H25	120.8
C26—C21—C2	117.08 (16)	C46—C45—C44	117.67 (17)
C22—C21—C2	124.25 (18)	C46—C45—H45	121.2
O2—C26—C21	121.37 (16)	C44—C45—H45	121.2
O2—C26—C25	116.34 (18)	C45—C46—C41	120.74 (18)
C21—C26—C25	122.28 (17)	C45—C46—N1	129.92 (16)
C5—C6—O1	123.59 (16)	C41—C46—N1	109.34 (15)
C5—C6—C61	122.55 (17)	C44—C43—C42	118.42 (18)
O1—C6—C61	113.81 (15)	C44—C43—C48	121.13 (18)
C65—C66—C61	119.73 (19)	C42—C43—C48	120.45 (18)
C65—C66—H66	120.1	C25—C24—C23	120.9 (2)
C61—C66—H66	120.1	C25—C24—H24	119.5
O3—C31—O2	116.87 (17)	C23—C24—H24	119.5
O3—C31—C3	125.33 (17)	C43—C48—H48A	109.5
O2—C31—C3	117.78 (15)	C43—C48—H48B	109.5
C3—C2—O1	123.50 (16)	H48A—C48—H48B	109.5
C3—C2—C21	122.19 (17)	C43—C48—H48C	109.5
O1—C2—C21	114.31 (15)	H48A—C48—H48C	109.5
C2—C3—C31	118.98 (16)	H48B—C48—H48C	109.5
C2—C3—C4	123.23 (17)	C63—C64—C65	121.23 (19)
C31—C3—C4	117.76 (15)	C63—C64—H64	119.4
C23—C22—C21	119.4 (2)	C65—C64—H64	119.4
C23—C22—H22	120.3		
			
C6—C5—C4—C41	119.26 (19)	O3—C31—C3—C4	5.5 (3)
C51—C5—C4—C41	−59.0 (2)	O2—C31—C3—C4	−173.18 (16)
C6—C5—C4—C3	−4.6 (2)	C5—C4—C3—C2	7.4 (2)
C51—C5—C4—C3	177.16 (15)	C41—C4—C3—C2	−118.02 (19)
C6—C5—C4—C47	−127.26 (18)	C47—C4—C3—C2	129.91 (19)
C51—C5—C4—C47	54.5 (2)	C5—C4—C3—C31	−174.52 (16)
C62—O4—C51—O5	179.51 (17)	C41—C4—C3—C31	60.1 (2)
C62—O4—C51—C5	0.0 (3)	C47—C4—C3—C31	−52.0 (2)
C6—C5—C51—O5	178.95 (18)	C26—C21—C22—C23	0.8 (3)
C4—C5—C51—O5	−2.7 (3)	C2—C21—C22—C23	−179.77 (19)
C6—C5—C51—O4	−1.6 (3)	C46—N1—C47—O6	−179.44 (19)
C4—C5—C51—O4	176.77 (15)	C46—N1—C47—C4	−0.5 (2)
C51—O4—C62—C63	−176.75 (17)	C5—C4—C47—O6	58.0 (2)
C51—O4—C62—C61	2.6 (3)	C41—C4—C47—O6	179.11 (18)
C66—C61—C62—O4	178.32 (17)	C3—C4—C47—O6	−62.8 (2)
C6—C61—C62—O4	−3.4 (3)	C5—C4—C47—N1	−120.92 (16)
C66—C61—C62—C63	−2.4 (3)	C41—C4—C47—N1	0.15 (19)
C6—C61—C62—C63	175.88 (18)	C3—C4—C47—N1	118.29 (16)
C31—O2—C26—C21	1.1 (3)	C5—C4—C41—C42	−61.1 (3)
C31—O2—C26—C25	−178.23 (18)	C3—C4—C41—C42	61.1 (3)
C22—C21—C26—O2	179.75 (18)	C47—C4—C41—C42	179.55 (19)
C2—C21—C26—O2	0.3 (3)	C5—C4—C41—C46	119.56 (17)
C22—C21—C26—C25	−0.9 (3)	C3—C4—C41—C46	−118.15 (17)
C2—C21—C26—C25	179.66 (19)	C47—C4—C41—C46	0.26 (19)
C51—C5—C6—O1	178.11 (16)	C21—C22—C23—C24	−0.1 (3)
C4—C5—C6—O1	−0.1 (3)	C61—C66—C65—C64	1.0 (3)
C51—C5—C6—C61	0.6 (3)	C46—C41—C42—C43	1.6 (3)
C4—C5—C6—C61	−177.62 (17)	C4—C41—C42—C43	−177.67 (18)
C2—O1—C6—C5	2.8 (3)	O4—C62—C63—C64	−178.62 (19)
C2—O1—C6—C61	−179.47 (15)	C61—C62—C63—C64	2.1 (3)
C62—C61—C6—C5	1.8 (3)	O2—C26—C25—C24	179.55 (19)
C66—C61—C6—C5	179.99 (18)	C21—C26—C25—C24	0.2 (3)
C62—C61—C6—O1	−175.87 (16)	C43—C44—C45—C46	0.6 (3)
C66—C61—C6—O1	2.3 (3)	C44—C45—C46—C41	−0.2 (3)
C62—C61—C66—C65	0.8 (3)	C44—C45—C46—N1	178.69 (19)
C6—C61—C66—C65	−177.36 (19)	C42—C41—C46—C45	−0.9 (3)
C26—O2—C31—O3	177.49 (18)	C4—C41—C46—C45	178.49 (17)
C26—O2—C31—C3	−3.8 (3)	C42—C41—C46—N1	−179.95 (17)
C6—O1—C2—C3	0.1 (3)	C4—C41—C46—N1	−0.6 (2)
C6—O1—C2—C21	−179.93 (16)	C47—N1—C46—C45	−178.25 (19)
C26—C21—C2—C3	1.1 (3)	C47—N1—C46—C41	0.7 (2)
C22—C21—C2—C3	−178.30 (18)	C45—C44—C43—C42	0.1 (3)
C26—C21—C2—O1	−178.86 (16)	C45—C44—C43—C48	179.5 (2)
C22—C21—C2—O1	1.7 (3)	C41—C42—C43—C44	−1.1 (3)
O1—C2—C3—C31	176.20 (17)	C41—C42—C43—C48	179.48 (19)
C21—C2—C3—C31	−3.8 (3)	C26—C25—C24—C23	0.6 (3)
O1—C2—C3—C4	−5.7 (3)	C22—C23—C24—C25	−0.7 (4)
C21—C2—C3—C4	174.32 (17)	C62—C63—C64—C65	−0.2 (3)
O3—C31—C3—C2	−176.4 (2)	C66—C65—C64—C63	−1.3 (3)
O2—C31—C3—C2	5.0 (3)		

### Hydrogen-bond geometry (Å, º)

**Table:**

*D*—H···*A*	*D*—H	H···*A*	*D*···*A*	*D*—H···*A*
N1—H1···O5^i^	0.89 (3)	2.25 (3)	2.965 (2)	138 (2)
C45—H45···O5^i^	0.93	2.51	3.212 (2)	133
C63—H63···O3^ii^	0.93	2.51	3.347 (3)	150
C65—H65···O4^iii^	0.93	2.59	3.363 (2)	141

Symmetry codes: (i) −*x*+1, −*y*, −*z*; (ii) −*x*+1, *y*+1/2, −*z*+1/2; (iii) *x*, −*y*+1/2, *z*+1/2.
